# Retraction Notice to: The Potential Therapeutic Role of Exosomal MicroRNA-520b Derived from Normal Fibroblasts in Pancreatic Cancer

**DOI:** 10.1016/j.omtn.2022.07.011

**Published:** 2022-07-14

**Authors:** Huijuan Shi, Hui Li, Tiantian Zhen, Yu Dong, Xiaojuan Pei, Xiangliang Zhang

## Main text

(Molecular Therapy: Nucleic Acids *20*, 373–384; June 2020)

This article has been retracted at the request of the editors.

*Molecular Therapy – Nucleic Acids* is retracting this article. Concerns regarding data integrity were raised by the National Science Library of the Chinese Academy of Sciences, which were based on a PubPeer thread showing similarities among western blots and an illustration in this article and articles published by unrelated authors, as well as questions regarding the expression of miR-520b and methods used for isolation and identification of exosomes (https://pubpeer.com/publications/EAA8DAF044ECE03E9B3FE6F8386630). The editors reached out to the authors to request the original data. Per journal policy, the editors, at their discretion, may request deposition of any or all original data files for examination by the reviewers and/or editors. As a result of the authors’ failure to provide these data—in violation of journal policy—and the allegations of fraud, the editors feel that findings of the manuscript cannot be relied upon. All authors have been informed.

